# U-shaped association between serum chloride and hypertension risk with nadir around 103 mmol/L: insights from regression and interpretable machine learning (XGBoost/SHAP) using NHANES 2017-2018

**DOI:** 10.3389/fphys.2025.1612895

**Published:** 2025-06-24

**Authors:** Shancheng He, Xuemei Zhong, Guangming Chen, Long Li

**Affiliations:** ^1^ Ganzhou Key Laboratory of Respiratory Diseases, Department of Intensive Care Medicine, Ganzhou Fifth People’s Hospital, Ganzhou Institute of Respiratory Disease Prevention and Control, Ganzhou, Jiangxi, China; ^2^ Department of Quality Control, Second People’s Hospital of Nankang District, Ganzhou, Jiangxi, China

**Keywords:** serum chloride, NHANES, hypertension, multiple regression analysis, smooth curve fitting

## Abstract

**Background:**

Hypertension is a major contributing factor for cardiovascular disease. This research attempted to explicate the link between serum levels of chloride and the risk of hypertension occurrence, as well as to identify the threshold level at which the risk undergoes changes across the general population and various demographic segments.

**Methods:**

Employing materials from the to 2017–2018 National Health and Nutrition Examination Survey (NHANES; n = 4,591), we employed multivariate regression analysis to gauge the connection between adult serum chloride concentrations and the risk of hypertension occurring. Smooth curve fitting, threshold effects, and saturation effects analyses were carried out to identify the threshold levels of chloride connected with changes in the risk of hypertension. Additionally, to further explore the complex relationship between serum chloride levels and hypertension risk, and to understand the contributions of various features within a high-performance machine learning model, we trained an XGBoost classifier to predict hypertension status and utilized SHAP (SHapley Additive exPlanations) values for interpretation.

**Results:**

A substantial connection was acquired between serum chloride levels and the risk of hypertension. After adjusting for variables, the multivariate logistic regression analysis demonstrated a U-shaped connection (OR = 0.94, 95% CI: 0.92–0.97, P < 0.0001). Below 103 mmol/L, the risk of hypertension decreased with increasing chloride levels (OR = 0.906, 95% CI = 0.877–0.936, P < 0.0001), demonstrating a 9.4% decline in the likelihood of hypertension per 1 mmol/L rise in chloride. Conversely, above 103 mmol/L, the risk grew with higher chloride concentrations (OR = 1.119, 95% CI = 1.030–1.216, P = 0.0081), signifying an 11.9% rise in the probability of hypertension by 1 mmol/L increment. Interpretation of an XGBoost machine learning model using SHAP values visually corroborated this U-shaped pattern, further indicating that the lowest contribution of serum chloride to the predicted risk of hypertension occurred around the 103 mmol/L level.

**Conclusion:**

In conclusion, using NHANES 2017–2018 data, this study revealed a significant U-shaped association between adult serum chloride levels and hypertension risk, with a nadir at 103 mmol/L. Both low and high chloride levels correlated with increased hypertension risk. This suggests serum chloride could serve as a potential biomarker for hypertension risk stratification, warranting further validation. Given the observational design, future prospective studies are needed to confirm this association and elucidate its underlying mechanisms.

## 1 Introduction

Hypertension is a contributing risk for cardiovascular disease and poses a threat to global health (Zhou et al., 2021; Olsen et al., 2016). Chronic hypertension can lead to macrovascular conditions such as aortic dissection, heart failure, and stroke, as well as microvascular diseases such as kidney disease and retinopathy (Xie et al., 2016; Sharp et al., 2011; Cipolla et al., 2018; Hibino et al., 2022; Comeau et al., 2022). Excessive blood pressure is a crucial preventable driver of cardiovascular mortality and cardiovascular disease burden in most locations around the world (Zhou et al., 2021; Olsen et al., 2016).

Chloride ions, once thought to be inert in physiological processes, are now understood to be dynamically regulated, with intracellular fluctuations participating in a myriad of physiological activities (Yang et al., 2012; Wang et al., 2006). Serum chloride levels correlate with a spectrum of diseases, including pulmonary arterial hypertension (Sinha et al., 2022), vascular calcification (Hu et al., 2021; Zhao et al., 2012), heart disease (Grodin et al., 2015), chronic renal failure (Khatri et al., 2020), cardiorenal syndrome (Kazory and Costanzo, 2020), and hypertension (Sinha et al., 2022). Substantial evidence suggests that the increase in arterial pressure caused by salt ingestion is more precisely connected to the anionic component, chloride ions, than to sodium ions (Wiig et al., 2013; McCallum et al., 2013). The study of the correlation between serum chloride concentrations and hypertension has significant implications. Therefore, this study aimed to utilize data from the NHANES 2017-2018 to precisely characterize the association between serum chloride levels and the risk of hypertension, identify potential non-linear relationships and critical thresholds, and explore these dynamics across different demographic groups.

## 2 Participants and methods

### 2.1 Source of data and research group

**FIGURE 1:**
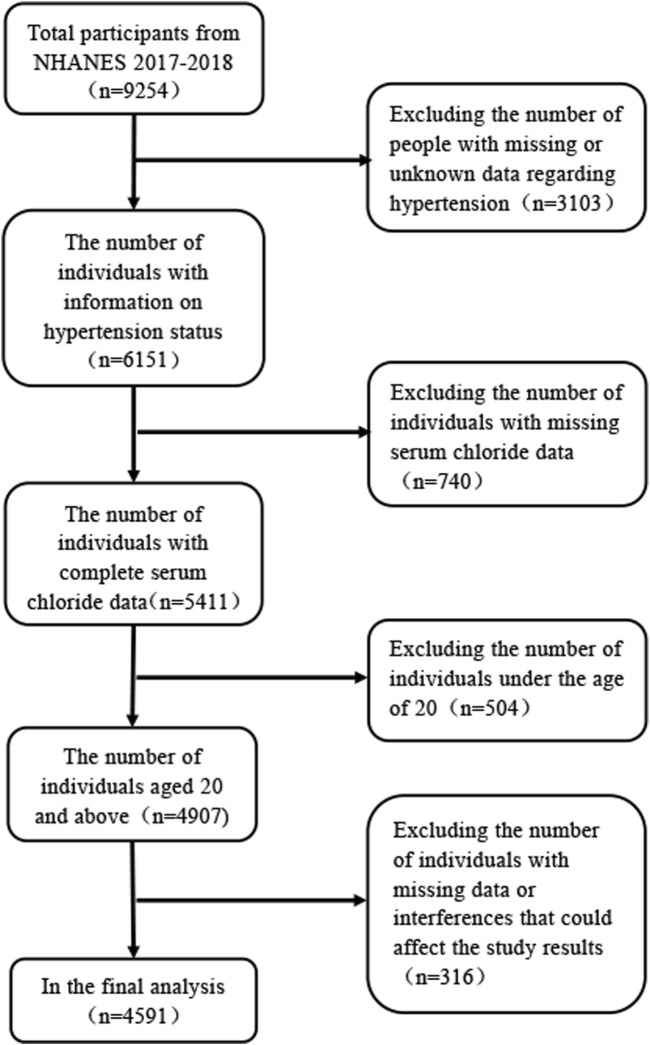
Flowchart of participant selection from NHANES 2017-2018.

The data employed in the analyses was gathered from the National Health and Nutrition Examination Survey (NHANES) database, which is renowned for its rigor and reliability, as substantiated by numerous studies. The NHANES is designed to collate data on physical health indicators, lifestyle, and dietary habits of the American populace to appraise their wellbeing. Our analysis was predicated on data recorded between 2017 and 2018, encompassing a single cycle within the NHANES database. The study initially comprised 9,255 individuals. During the data curation process, we excluded individuals with missing or unknown hypertension status, those under the age of 20 years, and individuals with incomplete data regarding age, ethnicity, marital status, height, weight, serum chloride, blood urea nitrogen, uric acid, potassium, and sodium levels, as well as those who might confound the study outcomes (Figure 1).

### 2.2 Variables

In the current research, the exposure variable was serum chloride (mmol/L), and its concentration was measured utilizing ion-selective electrodes that generated a potential based on the unique properties of certain membranes that come into contact with a diluted (1:31) solution containing chloride ions to determine its concentration. Serum chloride concentrations were classified into four groups: quartile 1 (Q1; ≥84, ≤101 mmol/L) (n = 2,592), quartile 2 (Q2; >101, ≤103 mmol/L) (n = 1,179), quartile 3 (Q3; >103, ≤105 mmol/L) (n = 599), and quartile 4 (Q4; >105, ≤112 mmol/L) (n = 221). These categories were determined based on prior observations of serum chloride in relation to other diseases (Hu et al., 2021; Zhao et al., 2024; Hou et al., 2023). The outcome variable was the occurrence of hypertension, which was assessed by Whether a doctor or another healthcare professional had told the individual of their hypertension through questionnaires in the NHANES database. In order to make this study more rigorous and precise, we have added 18 covariates: marital status, race/hispanic origin, smoking status, sex, height (feet), albumin (g/L), urea nitrogen (mmol/L), age, uric acid (umol/L), lactate dehydrogenase (IU/L), weight (pounds), blood sodium (mmol/L), triglycerides (mmol/L), blood calcium (mmol/L), creatinine (umol/L), blood potassium (umol/L), bicarbonate (mmol/L), and blood phosphorus (mmol/L). For all data on the exposure variable, outcome variable, and other variables, please browse to https://www.cdc.gov/nchs/nhanes/.

### 2.3 Statistical analysis

Each analysis was conducted utilizing the data analysis packages in R and Empower Statistics. Statistical worthiness was set at P < 0.05. To evaluate differences in descriptive analysis, t-tests (for continuous variables) or chi-square tests (for categorical variables) were utilized in the statistical procedure. Univariate analysis was utilized to gauge factors affecting hypertension, while a multivariate logistic regression model was utilized to gauge the connection between serum chloride concentrations and the risk of hypertension. The regression analysis produced three statistical models. Model I was not adjusted for these variables. Model II was adjusted for variables, such as race/Hispanic origin, age, and sex. Model III was adjusted for all relevant variables, such as blood urea nitrogen, marital status, albumin, race/Hispanic origin, age, uric acid, lactate dehydrogenase, weight, triglycerides, blood calcium, sex, creatinine, and bicarbonate. Smooth curve fitting and generalized additive models were utilized to evaluate the non-linear connection between chloride concentrations and the risk of hypertension occurring. To further explore the complex relationship between serum chloride levels and hypertension risk, and to understand the contributions of various features within a high-performance machine learning model, we trained an XGBoost classifier to predict hypertension status and utilized SHAP (SHapley Additive exPlanations) values for interpretation. The dataset (n = 4,591) was randomly split into a 70% training set (n = 3,213) and a 30% testing set (n = 1,378) using stratified sampling. During training, we employed 5-fold cross-validation for hyperparameter tuning and robust evaluation. The model’s predictive performance was assessed by calculating standard metrics (including Accuracy, Area Under the ROC Curve (AUC-ROC), Precision, Recall, and F1-Score) on the test set. For model interpretation, we utilized SHAP (SHapley Additive exPlanations) values and generated SHAP dependence plots to visualize feature contributions.

## 3 Results

An aggregate of 4,591 individuals was enrolled and categorized into quartiles based on serum chloride concentrations: quartile 1 (Q1; n = 2,592), quartile 2 (Q2; n = 1,179), quartile 3 (Q3; n = 599), and quartile 4 (Q4; n = 221). As depicted in Table 1, the mean serum chloride levels for the four groups were 99.07 ± 2.07 mmol/L, 102.43 ± 0.50 mmol/L, 104.36 ± 0.48 mmol/L, and 106.63 ± 0.98 mmol/L, respectively. Significant baseline differences were observed across the quartiles of serum chloride for smoking status, body weight, sex, albumin, blood urea nitrogen, uric acid, race/Hispanic ethnicity, blood phosphorus, lactate dehydrogenase, triglycerides, blood calcium, height, bicarbonate, blood potassium, age, and blood sodium. In contrast, no significant baseline differences were noted for marital status or creatinine.

**TABLE 1:** Baseline characteristics of participants (N = 4,591).

Serum Chloride (mmol/L) Tertile	Quartile 1 (≥84,≤101)	Quartile2 (>101,≤103)	Quartile3 (>103,≤105)	Quartile4 (>105,≤112)	p
N	2,592	1,179	599	221	
Serum chloride (mmol/L)	99.07 ± 2.07	102.43 ± 0.50	104.36 ± 0.48	106.63 ± 0.98	
Age (years)	52.10 ± 17.42	50.05 ± 17.93	52.32 ± 17.91	53.20 ± 17.82	0.003
Height (feet)	66.40 ± 4.14	65.96 ± 4.22	65.91 ± 4.27	65.90 ± 4.01	0.003
Weight (pounds)	181.15 ± 49.74	178.65 ± 46.10	184.12 ± 47.39	187.49 ± 48.75	0.023
Albumin (g/L)	41.08 ± 3.33	39.85 ± 3.38	39.17 ± 3.06	38.48 ± 3.03	<0.001
Blood Urea Nitrogen (mmol/L)	5.42 ± 2.16	5.33 ± 2.14	5.32 ± 1.99	5.76 ± 2.61	0.038
Lactate Dehydrogenase (IU/L)	158.32 ± 33.11	158.94 ± 32.58	162.41 ± 34.04	164.80 ± 38.02	0.004
Uric Acid (μmol/L)	330.75 ± 89.68	316.94 ± 83.87	320.57 ± 91.41	321.57 ± 94.13	<0.001
Triglycerides (mmol/L)	1.66 ± 1.09	1.51 ± 1.02	1.38 ± 0.76	1.42 ± 0.73	<0.001
Creatinine (μmol/L)	80.77 ± 43.47	78.83 ± 28.05	78.37 ± 24.11	84.66 ± 33.86	0.080
Potassium (mmol/L)	4.03 ± 0.37	4.10 ± 0.37	4.18 ± 0.35	4.23 ± 0.35	<0.001
Sodium (mmol/L)	139.09 ± 2.49	141.17 ± 2.12	142.53 ± 2.10	143.89 ± 2.22	<0.001
Bicarbonate (mmol/L)	25.98 ± 2.51	25.50 ± 2.44	25.10 ± 2.38	24.36 ± 2.44	<0.001
Phosphorus (mmol/L)	1.16 ± 0.16	1.14 ± 0.16	1.12 ± 0.17	1.11 ± 0.16	<0.001
Calcium (mmol/L)	2.34 ± 0.09	2.31 ± 0.08	2.30 ± 0.10	2.27 ± 0.09	<0.001
Gender					<0.001
Male	1,340 (51.70%)	513 (43.51%)	249 (41.57%)	98 (44.34%)	
Female	1,252 (48.30%)	666 (56.49%)	350 (58.43%)	123 (55.66%)	
Race/Hispanic Origin					
Mexican American	316 (12.19%)	151 (12.81%)	79 (13.19%)	26 (11.76%)	0.013
Other Hispanic	236 (9.10%)	96 (8.14%)	54 (9.02%)	22 (9.95%)	
Non-Hispanic White	955 (36.84%)	430 (36.47%)	194 (32.39%)	82 (37.10%)	
Non-Hispanic Black	558 (21.53%)	278 (23.58%)	176 (29.38%)	58 (26.24%)	
Other Races	527 (20.33%)	224 (19.00%)	96 (16.03%)	33 (14.93%)	
Marital Status					0.280
Married	1,337 (51.58%)	583 (49.45%)	277 (46.24%)	103 (46.61%)	
Widowed	203 (7.83%)	101 (8.57%)	54 (9.02%)	21 (9.50%)	
Other	1,052 (40.59%)	495 (41.98%)	268 (44.74%)	97 (43.89%)	
Smoking					<0.001
Yes	1,104 (42.59%)	485 (41.14%)	245 (40.90%)	123 (55.66%)	
No	1,488 (40.59%)	694 (58.86%)	354 (59.10%)	98 (44.34%)	
Hypertension					<0.001
Yes	1,524 (58.80%)	769 (65.22%)	389 (64.94%)	122 (55.20%)	
No	1,068 (41.20%)	410 (34.78%)	210 (35.06%)	99 (44.80%)	

Note: Continuous variables were expressed as mean ± standard deviation; and categorical variables were expressed as n (%).

Table 2 revealed the outcomes of the univariate analysis, indicating a link between serum chloride concentrations and the likelihood of developing hypertension. In the analysis of the risk of hypertension, utilizing Q1 as the comparison group, the odds ratios (OR) for Q2 (OR = 0.76, 95% CI: 0.66-0.88, P = 0.0002) and Q3 (OR = 0.77, 95% CI: 0.64-0.93, P = 0.0057) were both less than 1. In contrast, Q4 showed no statistical significance compared to Q1 (P > 0.05). The likelihood of hypertension occurrence was additionally connected with marital status, weight, blood urea nitrogen, race, lactate dehydrogenase, uric acid, triglycerides, smoking status, creatinine, age, blood calcium, serum albumin, and bicarbonate; however, blood potassium, height, blood sodium, sex, and blood phosphorus were not related to the likelihood of hypertension. To further investigate, were utilized subgroup analyses to gauge the various connections between serum chloride quartiles and the likelihood of hypertension in the subgroups (shown in Table 3). Significant interactions between plasma chloride concentration and the likelihood of hypertension occurring were observed in subgroups defined by sex, lactate dehydrogenase, creatinine, and blood sodium (P-value for interaction <0.05). In the female subgroup, the Q3 and Q2 populations had a lower likelihood of hypertension than the Q1 population. In the subgroup categorized by blood sodium levels, the Q2 and Q3 groups had a lower likelihood of hypertension, with the Q1 group serving as a comparison group, whereas the Q4 group had a higher likelihood of hypertension occurring.

**TABLE 2:** Univariate analysis of the risk of hypertension occurrence.

Variable	Statistics	Hypertension OR (95%CI) P-value
Serum Chloride (mmol/L)	100.99 ± 2.86	0.93 (0.91,0.95)<0.0001
Serum Chloride (mmol/L) Quartile		
Quartile 1	2,592 (56.46%)	1.0
Quartile 2	1,179 (25.68%)	0.76 (0.66,0.88) 0.0002
Quartile 3	599 (13.05%)	0.77 (0.64,0.93)0.0057
Quartile 4	221 (4.81%)	1.16 (0.88,1.53)0.2984
Albumin (g/L)	40.39 ± 3.40	0.95 (0.93,0.96) <0.001
Albumin (g/L) Tertile		
Low	1,220 (26.57%)	1.0
Medium	1,639 (36.70%)	0.79 (0.68,0.92) 0.0020
High	1732 (37.73%)	0.62 (0.54,0.72)<0.0001
Blood Urea Nitrogen (mmol/L)	5.402 ± 2.159	1.32 (1.28,1.37)<0.0001
Blood Urea Nitrogen (mmol/L) Tertile		
Low	1,239 (26.99%)	1.0
Medium	1,578 (34.37%)	1.41 (1.19,1.66) <0.0001
High	1774 (38.64%)	3.02 (2.58,3.53)<0.0001
Lactate Dehydrogenase (IU/L)	159.33 ± 33.39	1.01 (1.01,1.01) <0.0001
Lactate Dehydrogenase (IU/L) Tertile		
Low	1,469 (32.00%)	1.0
Medium	1,546 (33.68%)	1.25 (1.08,1.46)0.0034
High	1,576 (34.33%)	1.916 (1.653,2.221)<0.0001
Uric Acid (μmol/L)	325.43 ± 88.86	1.005 (1.004,1.006)<0.0001
Uric Acid (μmol/L) Tertile		
Low	1,461 (31.82%)	1.0
Medium	1,546 (33.67%)	1.49 (1.28,1.74)<0.0001
High	1,584 (34.50%)	2.51 (2.16,2.92)<0.0001
Triglycerides (mmol/L)	1.57 ± 1.02	1.25 (1.18,1.33)<0.0001
Triglycerides (mmol/L) Tertile		
Low	1,521 (33.13%)	1.0
Medium	1,529 (33.30%)	1.452 (1.25,1.69)<0.0001
High	1,541 (33.57%)	2.06 (1.77,2.39)<0.0001
Creatinine (μmol/L)	80.15 ± 37.43	1.02 (1.02,1.02)<0.0001
Creatinine (μmol/L) Tertile		
Low	1,514 (32.98%)	1.0
Medium	1,519 (33.09%)	1.31 (1.12,1.52)0.0005
High	1,558 (33.94%)	2.16 (1.86,12.50)<0.0001
Potassium (mmol/L)	4.08 ± 0.37	1.02 (0.87,1.19)0.8273
Potassium (mmol/L) Tertile		
Low	1,208 (26.31%)	1.0
Medium	1,579 (34.39%)	0.70 (0.60,0.82)<0.0001
High	1804 (39.29%)	0.89 (0.77,1.04)0.1333
Sodium (mmol/L)	140.30 ± 2.79	1.02 (1.00,1.04)0.0887
Sodium (mmol/L) Tertile		
Low	1,165 (25.38%)	1.0
Medium	1,253 (27.29%)	0.97 (0.82,1.14)0.7060
High	2,173 (47.33%)	1.12 (0.97,1.30)0.1301
Bicarbonate (mmol/L)	25.67 ± 2.51	1.06 (1.03,1.09)<0.0001
Bicarbonate (mmol/L) Tertile		
Low	1,433 (31.21%)	1.0
Medium	1,482 (32.28%)	1.04 (0.89,1.21)0.6165
High	1,676 (36.51%)	1.32 (1.14,1.53)0.0002
Phosphorus (mmol/L)	1.149 ± 0.165	0.97 (0.68,1.38)0.8506
Phosphorus (mmol/L) Tertile		
Low	1,274 (27.75%)	1.0
Medium	1715 (37.36%)	1.10 (0.95,1.28)0.2019
High	1,602 (34.89%)	1.04 (0.89,1.21)0.6280
Total Calcium (mmol/L)	2.32 ± 0.09	9.11 (4.75,17.49)<0.0001
Total Calcium (mmol/L) Tertile		
Low	1,179 (25.68%)	1.0
Medium	1,517 (33.04%)	1.18 (1.01,1.38)0.0432
High	1895 (41.28%)	1.59 (1.37,1.85)<0.0001
Gender		
Male	2,200 (47.92%)	1.0
Female	2,391 (52.08%)	0.91 (0.81,1.02)0.1199
Age (years)	51.657 ± 17.658	1.06 (1.05,1.06)<0.0001
Age (years) Tertile		
<60	1779 (38.75%)	1.0
≥60	2,812 (61.25%)	0.20 (0.18,0.23)<0.0001
Race/Hispanic Origin		
Mexican American	572 (12.46%)	1.0
Other Hispanic	408 (8.89%)	1.39 (1.06,1.83)0.0174
Non-Hispanic White	1,661 (36.18%)	1.75 (1.42,2.15)<0.0001
Non-Hispanic Black	1,070 (23.31%)	2.37 (1.90,2.95)<0.0001
Other Races(a)	880 (19.17%)	1.27 (1.01,1.60)0.0432
Marital Status		
Married	2,300 (50.10%)	1.0
Widowed	379 (8.26%)	2.64 (2.11,3.31)<0.0001
Other	1912 (41.65%)	0.72 (0.64,0.82)<0.0001
Smoking		
Yes	1957 (42.63%)	1.0
No	2,634 (57.37%)	0.66 (0.58,0.74)<0.0001
Height (feet)	66.20 ± 4.18	1.00 (0.98,1.01)0.8791
Height (feet) Tertile		
Low	1,302 (28.36%)	1.0
Medium	1,602 (34.89%)	0.92 (0.80,1.07)0.3027
High	1,687 (36.75%)	0.95 (0.82,1.10)0.5094
Weight (pounds)	184.76 ± 48.51	1.01 (1.01,1.01)<0.0001
Weight (pounds) Tertile		
Low	1,528 (33.28%)	1.0
Medium	1,522 (33.15%)	1.68 (1.45,1.95)<0.0001
High	1,541 (33.57%)	2.21 (1.91,2.57)<0.0001

Note: Continuous variables were expressed as mean ± standard deviation; and categorical variables were expressed as n (%). Abbreviation: CI, confidence interval. The first group was used as the reference for each univariate analysis group (OR, 1); (a) includes multiple races; weighted according to: full sample mobile examination center examination weights.

**TABLE 3:** Subgroup analysis of the risk of hypertension occurrence associated with serum chloride level.

Variable	Serum chloride				P Value for interaction
	Quartile1	Quartile2 OR (95%CI) P-value	Quartile3 OR (95%CI) P-value	Quartile4 OR (95%CI) P-value	
Gender					0.0184
Male	1.0	0.85 (0.69,1.05)0.1393	1.07 (0.81,1.40)0.6432	1.19 (0.79,1.80)0.4036	
Female	1.0	0.69 (0.57,0.84)0.0002	0.60 (0.46,0.77)<0.001	1.12 (0.77,1.63)0.5397	
Age (years)					0.7275
<60	1.0	0.85 (0.68,1.08)0.1886	0.70 (0.53,0.92)0.0108	1.08 (0.69,1.68)0.7342	
≥60	1.0	0.72 (0.59,0.89)0.0023	0.71 (0.54,0.95)0.0187	1.13 (0.76,1.69)0.5442	
Race/Hispanic Origin					0.3412
Mexican American	1.0	0.97 (0.63,1.49)0.8929	0.60 (0.33,1.09)0.0946	0.87 (0.35,2.14)0.7617	
Other Hispanic	1.0	0.46 (0.27,0.79)0.0044	0.61 (0.32,1.16)0.1348	1.01 (0.42,2.46)0.9832	
Non-Hispanic White	1.0	0.72 (0.57,0.91)0.0062	0.75 (0.55,1.03)0.0784	1.54 (0.98,2.42)0.0618	
Non-Hispanic Black	1.0	0.92 (0.69,1.23)0.5891	0.86 (0.61,1.21)0.3984	1.11 (0.65,1.91)0.7041	
Other Races	1.0	0.64 (0.46,0.91)0.0115	0.71 (0.44,1.14)0.1516	0.64 (0.29,1.41)0.2721	
Marital Status					0.6333
Married	1.0	0.71 (0.58,0.86)0.0007	0.72 (0.55,0.94)0.0162	0.90 (0.60,1.36)0.6280	
Widowed	1.0	0.65 (0.40,1.06)0.0858	0.70 (0.38,1.30)0.2587	1.20 (0.45,3.25)0.7131	
Other maritai status	1.0	0.84 (0.67,1.06)0.1519	0.84 (0.63,1.13)0.2492	1.51 (0.99,2.30)0.0545	
Smoking					0.4055
Yes	1.0	0.68 (0.55,0.85)0.0006	0.76 (0.58,1.01)0.0579	0.94 (0.65,1.36)0.7401	
No	1.0	0.83 (0.69,1.01)0.0621	0.78 (0.61,1.00)0.0548	1.32 (0.87,2.00)0.1879	
Height (feet) Tertile					0.6619
Low	1.0	0.67 (0.52,0.87)0.0030	0.70 (0.50,0.98)0.0355	0.93 (0.55,1.56)0.7793	
Middle	1.0	0.76 (0.60,0.97)0.0288	0.67 (0.49,0.94)0.0.0182	1.30 (0.84,2.04)0.2427	
High	1.0	0.83 (0.66,1.06)0.1335	0.93 (0.68,1.26)0.6271	1.21 (0.74,1.95)0.4481	
Weight (pounds) Tertile					0.9481
Low	1.0	0.80 (0.61,1.04)0.0951	0.75 (0.52,1.07)0.1121	0.93 (0.52,1.66)0.8048	
Middle	1.0	0.75 (0.59,0.96)0.0233	0.75 (0.55,1.03)0.0748	1.37 (0.86,2.17)0.1831	
High	1.0	0.75 (0.58,0.95)0.0195	0.76 (0.56,1.04)0.0838	1.00 (0.64,1.57)0.9921	
Albumin (g/L) Tertile					0.0591
Low	1.0	0.68 (0.52,0.89)0.0052	0.61 (0.45,0.84)0.0021	1.12 (0.73,1.71)0.5985	
Middle	1.0	0.69 (0.55,0.87)0.0019	0.54 (0.39,0.73)<0.0001	0.81 (0.51,1.26)0.3472	
High	1.0	0.72 (0.56,0.94)0.0137	1.18 (0.81,1.71)0.3868	0.97 (0.46,2.03)0.9306	
Blood Urea Nitrogen (mmol/L) Tertile					0.5740
Low	1.0	0.70 (0.52,0.96)0.0247	0.97 (0.66,1.42)0.8831	0.86 (0.46,1.60)0.6263	
Middle	1.0	0.75 (0.59,0.97)0.0287	0.63 (0.45,0.88)0.0063	1.23 (0.75,2.02)0.4049	
High	1.0	0.79 (0.63,0.99)0.0394	0.79 (0.59,1.06)0.1203	1.18 (0.77,1.81)0.4347	
Lactate Dehydrogenase (IU/L) Tertile					0.0461
Low	1.0	0.53 (0.40,0.69)<0.0001	0.66 (0.46,0.95)0.0249	0.79 (0.46,1.34)0.3785	
Middle	1.0	0.86 (0.67,1.10)0.2230	0.68 (0.49,0.95)0.0231	1.49 (0.89,2.50)0.1296	
High	1.0	0.89 (0.70,1.13)0.3290	0.87 (0.65,1.17)0.3636	1.17 (0.76,1.79)0.4868	
Uric Acid (μmol/L) Tertile					0.1028
Low	1.0	0.63 (0.47,0.83)0.0011	0.68 (0.48,0.98)0.0360	1.51 (0.93,2.46)0.0990	
Middle	1.0	0.75 (0.59,0.96)0.0208	0.71 (0.51,0.98)0.0400	0.93 (0.58,1.49)0.7532	
High	1.0	1.01 (0.79,1.29)0.9238	0.97 (0.71,1.31)0.827+	1.27 (0.78,2.06)0.3438	
Triglycerides (mmol/L) Tertile					0.4573
Low	1.0	0.69 (0.53,0.90)0.0057	0.99 (0.73,1.36)0.9625	1.27 (0.78,2.08)0.3378	
Middle	1.0	0.87 (0.68,1.12)0.2787	0.71 (0.51,0.98)0.0345	1.22 (0.77,1.96)0.3991	
High	1.0	0.82 (0.64,1.05)0.1118	0.77 (0.55,1.09)0.1386	1.10 (0.68,1.79)0.7019	
Creatinine (μmol/L) Tertile					0.0141
Low	1.0	0.64 (0.49,0.84)0.0011	0.53 (0.37,0.75)0.0004	0.78 (0.45,1.35)0.3721	
Middle	1.0	0.67 (0.52,0.86)0.0018	0.72 (0.52,1.00)0.0528	1.30 (0.78,2.15)0.3142	
High	1.0	0.96 (0.76,1.22)0.7519	1.14 (0.84,1.56)0.3983	1.27 (0.82,1.95)0.2829	
Potassium (mmol/L) Tertile					0.3928
Low	1.0	0.78 (0.59,1.03)0.0807	0.98 (0.62,1.54)0.9248	1.45 (0.70,3.01)0.3181	
Middle	1.0	0.64 (0.50,0.83)0.0007	0.61 (0.44,0.84)0.0026	0.99 (0.58,1.69)0.9694	
High	1.0	0.87 (0.69,1.09)0.2198	0.89 (0.67,1.16)0.3793	1.21 (0.83,1.74)0.3190	
Sodium (mmol/L) Tertile					0.0007
Low	1.0	0.51 (0.33,0.78)0.0020	0.64 (0.22,1.83)0.4025	0.94 (0.74,1.14)0.1231	
Middle	1.0	0.52 (0.39,0.69)<0.0001	0.53 (0.32,0.88)0.0145	1.22 (1.05,1.39)0.0117	
High	1.0	0.86 (0.70,1.06)0.1524	0.75 (0.59,0.94)0.0143	0.95 (0.70,1.30)0.7545	
Bicarbonate (mmol/L) Tertile					0.4311
Low	1.0	0.81 (0.62,1.05)0.1143	0.96 (0.71,1.31)0.7998	1.58 (1.07,2.34)0.0217	
Middle	1.0	0.80 (0.62,1.03)0.0849	0.73 (0.52,1.01)0.0602 .	1.18 (0.70,2.01)0.5312	
High	1.0	0.74 (0.58,0.93)0.0105	0.72 (0.51,1.01)0.0543	0.73 (0.39,1.38)0.3396	
Phosphorus (mmol/L) Tertile					0.9572
Low	1.0	0.71 (0.54,0.93)0.0130	0.78 (0.56,1.09)0.1427	1.11 (0.69,1.76)0.6729	
Middle	1.0	0.78 (0.62,0.98)0.0353	0.80 (0.59,1.08)0.1449	1.35 (0.87,2.11)0.1804	
High	1.0	0.79 (0.62,1.01)0.0602	0.73 (0.52,1.02)0.0619	0.97 (0.56,1.68)0.9055	
Total calcium (mmol/L) Tertile					0.4975
Low	1.0	0.70 (0.53,0.94)0.0184	0.75 (0.53,1.05)0.0942	1.00 (0.65,1.55)0.9957	
Middle	1.0	0.79 (0.61,1.01)0.0563	0.96 (0.69,1.32)0.7865	1.59 (0.99,2.55)0.0566	
High	1.0	0.92 (0.73,1.16)0.4850	0.78 (0.57,1.08)0.1376	1.67 (0.88,3.15)0.1156	

Note: Continuous variables are presented as mean ± standard deviation; and categorical variables are represented as n (%). Abbreviations: CI, confidence interval. The first group is used as the reference for each univariate analysis group (OR, 1); (a) includes multiple races; weighted according to the full-sample mobile examination center examination weights.

Table 4 revealed the outcomes of the multivariate regression analysis, which revealed a negative connection between serum chloride concentrations and the likelihood of hypertension occurrence in Model I (OR = 0.93, 95% CI: 0.91-0.95, P < 0.0001). This inverse connection persisted after adjusting for confounding factors in Models II (OR = 0.93, 95% CI: 0.91-0.96, P < 0.0001) and III (OR = 0.94, 95% CI: 0.92-0.97, P < 0.0001). Furthermore, in the analysis of the risk of hypertension, it was observed across all models that compared to the reference group Q1, both Q2 and Q3 groups exhibited a lower risk of hypertension, whereas Q4 showed no significant association with Q1 (P > 0.05 for all comparisons).

**TABLE 4:** Relationship between serum chloride and the risk of hypertension occurrence (multivariate regression analysis).

Results	Model I OR (95%CI) P-value	Model II OR (95%CI) P-value	Model III OR (95%CI) P-value
Total Serum Chloride (mmol/L)	0.93 (0.91,0.95)<0.0001	0.93 (0.91,0.96)<0.0001	0.94 (0.92,0.97)<0.0001
Total Serum Chloride (mmol/L) Quartile			
Quartile 1 (≥84, ≤101)	1.0	1.0	1.0
Quartile2 (>101, ≤103)	0.76 (0.66,0.88)0.0002	0.79 (0.67,0.93)0.0045	0.86 (0.72,1.02)0.0799
Quartile3 (>103, ≤105)	0.77 (0.64,0.93)0.0057	0.67 (0.55,0.83)0.0002	0.71 (0.57,0.89)0.0029
Quartile4 (>105, ≤112)	1.16 (088,1.53)0.2984	1.09 (0.80,1.49)0.5909	1.14 (0.82,1.60)0.4360

Note: Weighted according to the full-sample mobile examination center examination weights. Abbreviations: CI, confidence interval. Outcome variable:whetherhypertension occurred. Exposure variable: serum chloride level (mmol/L). Model I: variables not adjusted.

Model II: adjusted for sex, age, and race. Model III: adjusted for factors related to hypertension risk. Adjustment based on: Sex,age, race, marital status, smoking (yes or no), body weight, serum albumin, blood urea nitrogen, lactate dehydrogenase, uric acid, triglycerides, creatinine, total calcium, and bicarbonate, etc.

**FIGURE 2:**
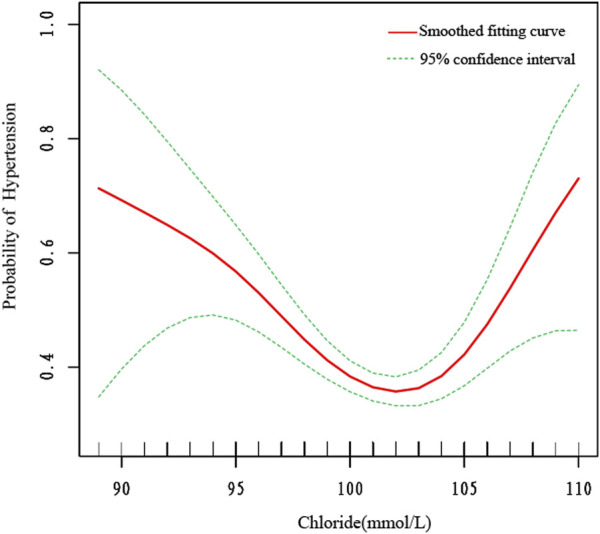
The smooth fitting curve delineating the relationship between serum chloride levels and the risk of hypertension occurrence. The smooth curve fitting is represented by the red line, and the fitted 95% confidence interval is represented by the green line. Weighting basis: Complete sample with mobile examination center examination weights. Adjusted for sex, age (smoothed), race, marital status, smoking status (yes or no), body weight (smoothed), serum albumin (smoothed), blood urea nitrogen (smoothed), lactate dehydrogenase (smoothed), uric acid (smoothed), triglycerides (smoothed), creatinine (smoothed), total calcium (smoothed), and bicarbonate (smoothed).

**FIGURE 3:**
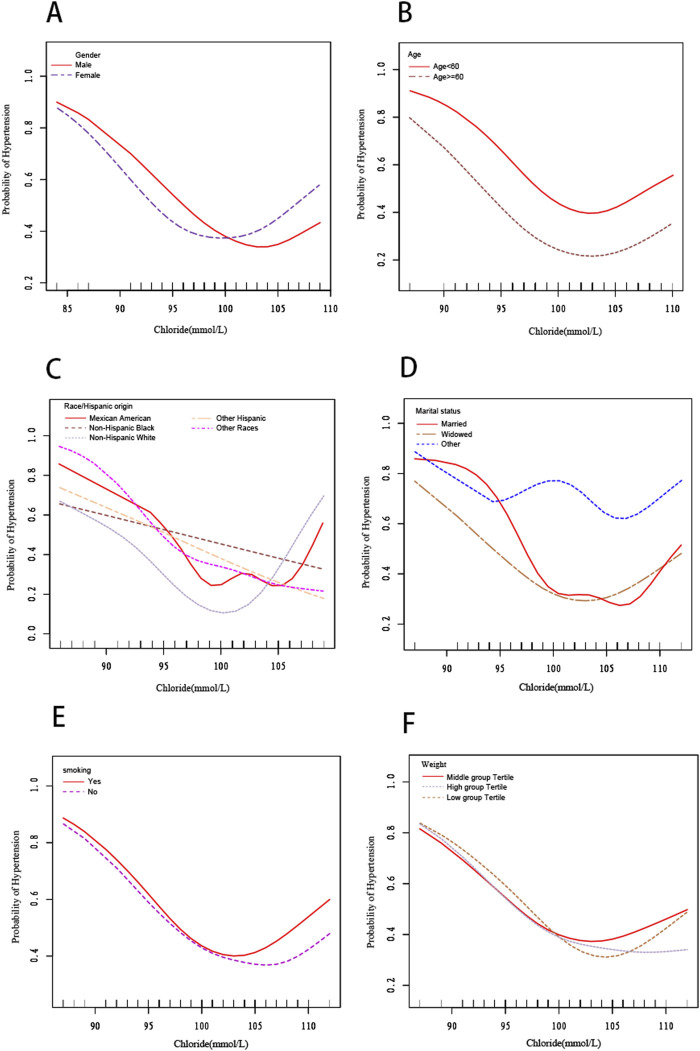
The smooth fitting curve delineating the relationship between serum chloride levels and the risk of hypertension occurrence, stratified by covariates (sex,age, race/Hispanic Origin, marital status, smoking status, weight). Adjusted for sex, age (smoothed), race, marital status, smoking status (yes or no), body weight (smoothed), serum albumin (smoothed), blood urea nitrogen (smoothed), lactate dehydrogenase (smoothed), uric acid (smoothed), triglycerides (smoothed), creatinine (smoothed), total calcium (smoothed), and bicarbonate (smoothed).**(A)** Stratified by sex. **(B)** Stratified by age. **(C)** Stratified by race/Hispanic origin. **(D)** Stratified by marital status. **(E)** Stratified by smoking status.**(F)** Stratified by weight (pounds).

We also utilized smooth curve fitting (Figure 2) and threshold and saturation effect analyses (Table 5) to gauge the link between serum chloride and the likelihood of hypertension occcurrence. For details on the covariates utilized for adjustment, see Table 5. Figure 2 illustrates that the fitted curve between chloride levels and the risk of hypertension exhibits a U-shaped pattern, initially declining and then rising, with a segmented effect:when chloride levels are below 103 mmol/L, the risk of hypertension decreases with increasing chloride concentrations (OR = 0.906, 95% CI: 0.877-0.936, P < 0.0001), with a 9.4% decline in the probability of developing hypertension for every 1 mmol/L increase in chloride; at chloride levels above 103 mmol/L, the risk of hypertension increases with increasing chloride levels (OR = 1.119, 95% CI: 1.030-1.216, P = 0.0081), with a 11.9% rise in the likelihood of developing hypertension (Figure 2; Table 5). To conduct a increasingly thorough analysis, smooth fitting curves were plotted for different strata of six covariates, including smoking status, age, body weight, race/Hispanic origin, sex, and marital status (Figure 3). In the majority of the stratified populations, the connection between serum chloride concentrations and the likelihood of hypertension occurrence has a segmented effect. Before the inflection point, the likelihood of hypertension occurring decreased with increasing chloride levels, and after the inflection point, the likelihood of hypertension occurrence rose with increasing chloride levels, with the smooth fitting curve exhibiting a U-shaped or U-shaped-like pattern. For instance, in the male population, the inflection point of the fitting curve corresponded to a serum chloride concentration of 98 mmol/L (see Table 5), and in the population younger than 60 years, the inflection point corresponded to a chloride concentration of 103 mmol/L (Table 5). The chloride concentrations corresponding to the inflection points of the fitting curves may not be the same. In other racial populations, the risk of hypertension declined with increasing chloride levels, whereas in Mexican-American, married, and widowed populations, the fitting curves exhibited considerable fluctuations.

**TABLE 5:** Threshold effect and saturation effect analysis.

Subgroup	Model I	Model II					
	A straight-line effect	Fold points (K)	< K-segment effect 1	>K-segment effect 2	Effect size difference of 2 *versus* 1	Equation predicted values at break points	Log likelihood ratio tests
Total							
Adjusted OR (95% CI)	0.945 (0.921,0.969)	103	0.906 (0.877,0.936)	1.119 (1.030,1.216)	1.235 (1.118,1.363)	−0.849 (-0.950,-0.749)	<0.001
*p*-value	<0.0001		<0.0001	0.0081	<0.0001		
Stratification variables							
*Age*							
<60 years							
Adjusted OR (95% CI)	0.930 (0.896,0.965)	103	0.880 (0.840,0.922)	1.158 (1.034,1.298)	1.316 (1.148,1.509)	−1.538 (-1.687,-1.389)	<0.001
*P*-value	0.0001		<0.0001	0.0113	<0.0001		
≥60 years							
Adjusted OR (95% CI)	0.941 (0.909,0.975)	104	0.910 (0.873,0.948)	1.219 (1.032,1.440)	1.340 (1.116,1.609)	0.130 (-0.035,0.295)	0.001
*P*-value	0.0007		<0.0001	0.0196	0.0017		
*Sex*							
*Male*							
Adjusted OR (95% CI)	0.957 (0.925,0.991)	98	0.816 (0.730,0.912)	1.003 (0.958,1.050)	1.230 (1.075,1.407)	−0.576 (-0.733,-0.418)	0.002
*P*-value	0.0127		0.0003	0.8876	0.0026		
*Female*							
Adjusted OR (95% CI)	0.934 (0.900,0.970)	104	0.897 (0.858,0.937)	1.280 (1.081,1.516)	1.428 (1.184,1.722)	−1.044 (-1.194,-0.893)	<0.001
*P*-value	0.0004		<0.0001	0.0042	0.0002		
*Race/ethnicity*							
Mexican American							
Adjusted OR (95% CI)	0.944 (0.874,1.018)	98	0.652 (0.482,0.881)	1.027 (0.931,1.133)	1.576 (1.113,2.232)	−1.048 (-1.396,-0.699)	0.007
*P*-value	0.1363		0.0053	0.5894	0.0103		
Other Hispanic							
Adjusted OR (95% CI)	0.918 (0.837,1.006)	105	0.890 (0.803,0.985)	1.664 (0.760,3.646)	1.871 (0.824,4.249)	−1.237 (-1.663,-0.811)	0.130
*P*-value	0.0684		0.0248	0.2029	0.1344		
Non-Hispanic White							
Adjusted OR (95% CI)	0.958 (0.921,0.997)	103	0.892 (0.847,0.938)	1.287 (1.126,1.472)	1.444 (1.231,1.693)	−0.882 (-1.051,-0.714)	<0.001
*P*-value	0.0336		<0.0001	0.0002	<0.0001		
Non-Hispanic Black							
Adjusted OR (95% CI)	0.972 (0.918,1.030)	103	0.964 (0.894,1.041)	0.998 (0.844,1.181)	1.035 (0.841,1.274)	−0.276 (-0.473,-0.078)	0.746
*P*-value	0.3420		0.3525	0.9820	0.7465		
Other races							
Adjusted OR (95% CI)	0.896 (0.841,0.954)	96	0.607 (0.382,0.964)	0.935 (0.868,1.007)	1.541 (0.946,2.511)	−0.481 (-0.815,-0.146)	0.029
*P*-value	0.0007		0.0344	0.07641	0.0825		
*Marital Status*							
*Married*							
Adjusted OR (95% CI)	0.937 (0.904,0.972)	96	0.552 (0.403,0.755)	0.978 (0.938,1.019)	1.772 (1.277,2.458)	−0.232 (-0.428,-0.037)	<0.001
*P*-value	0.0004		0.0002	0.2840	0.0006		
*Widowed*							
Adjusted OR (95% CI)	0.950 (0.879,1.027)	102	0.897 (0.802,1.003)	1.100 (0.892,1.357)	1.227 (0.935,1.609)	0.375 (0.052,0.699)	0.135
*P*-value	0.1961		0.0572	0.3721	0.1406		
*Other marital status*							
Adjusted OR (95% CI)	0.944 (0.906,0.984)	104	0.902 (0.859,0.947)	1.267 (1.063,1.511)	1.405 (1.153,1.712)	−1.223 (-1.399,-1.047)	<0.001
*P*-value	0.0060		<0.0001	0.0084	0.0007		
*Smoking*							
*Yes*							
Adjusted OR (95% CI)	0.933 (0.900,0.968)	103	0.884 (0.843,0.927)	1.133 (1.014,1.265)	1.282 (1.120,1.467)	−0.625 (-0.775,-0.475)	<0.001
*P*-value	0.0002		<0.0001	0.0275	0.0003		
*NO*							
Adjusted OR (95% CI)	0.959 (0.926,0.993)	103	0.928 (0.887,0.970)	1.118 (0.985,1.269)	1.205 (1.039,1.399)	−1.005 (-1.142,-0.868)	0.014
*P*-value	0.0202		0.0010	0.0843	0.0138		
*Weight (pounds) Tertile*							
*Low*							
Adjusted OR (95% CI)	0.942 (0.898,0.987)	97	0.765 (0.627,0.933)	0.984 (0.928,1.044)	1.287 (1.029,1.610)	−0.789 (-1.023,-0.556)	0.013
*P*-value	0.0126		0.0080	0.6013	0.0270		
*Middle*							
Adjusted OR (95% CI)	0.947 (0.906,0.989)	103	0.905 (0.855,0.958)	1.117 (0.974,1.281)	1.235 (1.047,1.457)	−0.779 (-0.951,-0.608)	0.012
*P*-value	0.0143		0.0006	0.1123	0.0124		
*High*							
Adjusted OR (95% CI)	0.937 (0.898,0.977)	103	0.882 (0.835,0.933)	1.167 (1.020,1.334)	1.322 (1.124,1.555)	−0.506 (-0.678,-0.333)	<0.001
*P*-value	0.0023		<0.0001	0.0242	0.0008		

Note: Abbreviations: CI, confidence interval. Weighted by: full-sample mobile examination center weights. The outcome variable of the study was the occurrence of hypertension, and the exposure variable is serum chloride level (measured in mmol/L). Adjustments were made for factors such as sex,age, race/Hispanic origin, marital status, smoking status (yes or no), body weight, albumin, blood urea nitrogen, lactate dehydrogenase, uric acid, triglycerides, creatinine, total calcium, and bicarbonate. When the P-value of the log-likelihood ratio test was less than 0.05, Model I is selected to represent the linear effect; when the P-value was greater than 0.05, Model II, was chosen to represent the segmented effect; and the Odds Ratio (OR) denotes the slope of the curve and is statistically significant for segments with a P-value less than 0.05. The K value refers to the breakpoint, that is, the specific level of blood chloride content at which the relationship between blood chloride and the risk of hypertension incidence changes.

**FIGURE 4:**
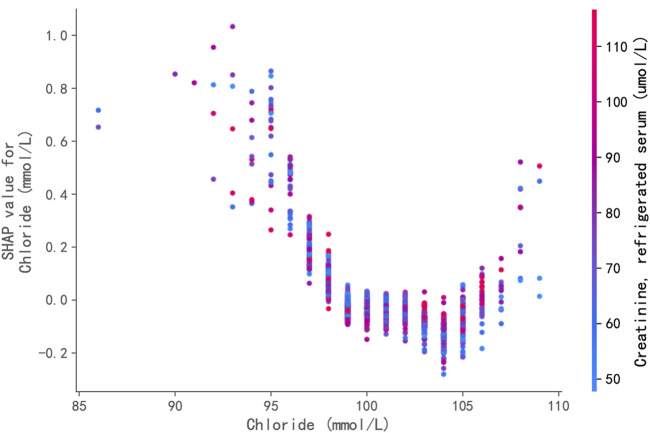
SHAP (SHapley Additive exPlanations) Dependence Plot for Serum Chloride. This plot displays the relationship between serum chloride levels (x-axis) and their impact (SHAP value, y-axis) on the XGBoost model’s prediction of hypertension risk. Each point represents an individual sample from the test set. Positive SHAP values indicate that the chloride level increased the model’s predicted likelihood of hypertension, while negative values indicate a decreased predicted likelihood. A distinct U-shaped non-linear relationship is evident: increasing chloride levels below ∼98 mmol/L are associated with decreasing risk contribution; levels between ∼98 and 103 mmol/L contribute minimally; and levels above ∼103 mmol/L are associated with a marked increase in positive contribution to the predicted risk.

The XGBoost classifier, trained to predict hypertension status, demonstrated good predictive capability on the independent test set. It achieved an overall accuracy of 72.1% and an AUC-ROC of 0.790. For predicting hypertension, the model yielded a precision of 0.66, a recall of 0.58, and an F1-score of 0.62. These metrics indicate that the model is sufficiently robust to support further feature contribution analysis using SHAP values. Figure 4 displays the SHAP dependence plot for serum chloride. Positive SHAP values indicate that a given chloride level increased the model’s predicted likelihood of hypertension, whereas negative values indicate a decreased predicted likelihood. Each point in the plot represents an individual sample from the test set. The plot reveals a distinct U-shaped (or potentially J-shaped) non-linear relationship between serum chloride levels and their contribution to the model’s prediction. Specifically, at lower serum chloride concentrations (approximately <98 mmol/L), increasing chloride levels were associated with a decreasing contribution (lower SHAP values) towards the predicted risk of hypertension, transitioning from positive towards zero or negative values. Within the intermediate range of approximately 98–103 mmol/L, the SHAP values were generally at their minimum, close to or below zero, suggesting that chloride levels in this range contributed minimally, or even negatively (protectively), to the predicted hypertension risk. Conversely, at higher serum chloride concentrations (approximately >103 mmol/L), further increases in chloride levels were associated with a marked increase in their positive contribution (increasing positive SHAP values) to the predicted risk.

## 4 Discussion

Essential hypertension develops because of complex interactions across several regulating mechanisms, which are effected by a variety of biological, nutritional, and environmental variables (McCallum et al., 2015; Cheung and Li, 2012). Studies on the effects of salt intake and potassium consumption on arterial pressure (Aung et al., 2023; Kou et al., 2023; Stolarz-Skrzypek et al., 2013), along with the pressure-natriuretic hypothesis (Guyton, 1991), indicated that sodium ions were the principal determinants of blood pressure and mortality, and chloride was the main extracellular anion that came from the diet accompanying sodium (Na) (Stolarz-Skrzypek et al., 2013). However, there was a strong indication in both animals and humans that the increase in arterial pressure due to salt consumption might be more closely related to the anionic component (i.e., Cl-) than to Na+ (Luft et al., 1990; Kotchen et al., 1983; Kurtz et al., 1987). Extensive studies on hypertensive rat models and humans have shown that equimolar additions of sodium salts can lead to similar extents of sodium storage and restriction of the renin-angiotensin-aldosterone system (RAAS), but only sodium chloride results in a volume of plasma growth and increased blood pressure (Kotchen et al., 1983; Kurtz et al., 1987). A 35-year epidemiological study of 12,968 hypertensive adults, measuring blood pressure and electrolytes longitudinally, revealed that low blood chloride levels were connected with higher cardiovascular mortality, irrespective of serum sodium or bicarbonate levels. A study involving 162 patients found that hypochloremia was significantly reduced regardless of whether or not it was accompanied by hyponatremia (Hanberg et al., 2016). The Belgian Interuniversity Research on Nutrition and Health, involving 9,106 subjects, discovered that after adjusting for sex,body mass index and other factors such as serum sodium, a chloride concentration of <100 mEq/L was correlated with an elevated susceptibility to a broad spectrum of diseases and a heightened risk of cardiovascular disease (Kurtz et al., 1987).

A wealth of current evidence indicates that chloride ions are not passive entities in the electrochemical equilibrium of the cytoplasmic membrane; they are dynamically regulated and play crucial roles in processes such as cell death (McCallum et al., 2015), modulation of enzymatic function (Cheung and Li, 2012), anticancer drug resistance (Aung et al., 2023), and synaptic transmission (Kou et al., 2023). Research has demonstrated that a decline in chloride transport by the macula densa can result in elevated renin secretion from the juxtaglomerular apparatus (Stolarz-Skrzypek et al., 2013), resulting in an active renin-angiotensin-aldosterone system, increased resistance in the renal afferent arterioles, lower renal circulation and glomerular filtration rate, and subsequently higher blood pressure (Stolarz-Skrzypek et al., 2013). Additional analyses revealed that low chloride levels in the thick ascending limb of the circle of Henle and the distal convoluted tubule could improve the activity of Na-K-Cl cotransporters through the lysine-less protein kinase family of intracellular chloride ion sensors (Guyton, 1991). Overactivity of these transporters can lead to increased sodium absorption, resistance to diuretics, and fluid overload (Guyton, 1991).

The primary goal of the investigation was to obtain a peak understanding at the connection between chloride and the likelihood of hypertension occurring. We utilized a sample with adequate representation of the adult population in America (n = 4,591). Our research has an array of advantages: (Zhou et al., 2021): a large sample size and a diverse study sample; (Olsen et al., 2016); rigorous statistical methods that take into account a wide range of potential factors in order to reduce the impact of confounding variables. This study suggests that serum chloride could serve as a population-level biomarker for stratifying hypertension risk, warranting further validation.

This study has several limitations. The individuals that we picked were all US citizens, therefore, the outcomes might vary in different nations and locations. This study measured chloride levels at a single time point; therefore, we could not assess the temporal connection between chloride levels and the likelihood of hypertension. Consequently, future studies should include long-term follow-up research. Furthermore, while the sample size of this study is substantial, data from additional individuals will strengthen the conclusions. The molecular pathway by which chloride regulates the occurrence of hypertension is unexplained and entails further basic research. Although we utilized statistical tools to control for confounding factors, we may still be unable to rule out the presence of other confounding factors.

## 5 Conclusion

In conclusion, using NHANES 2017–2018 data, this study revealed a significant U-shaped association between adult serum chloride levels and hypertension risk, with a nadir at 103 mmol/L. Both low and high chloride levels correlated with increased hypertension risk. This suggests serum chloride could serve as a potential biomarker for hypertension risk stratification, warranting further validation. Given the observational design, future prospective studies are needed to confirm this association and elucidate its underlying mechanisms.

## Data Availability

Publicly available datasets were analyzed in this study. This data can be found here: https://www.niehs.nih.gov/research/atniehs/labs/crb/studies/nhanes.
